# Non-muscle myosin heavy chain IIA regulates cell morphology, stress fibre structure, and cell migration in FLO-1 oesophageal adenocarcinoma cells

**DOI:** 10.1007/s13577-025-01196-w

**Published:** 2025-03-31

**Authors:** Deirdre Duff, Siobhan Gargan, Aideen Long

**Affiliations:** Trinity Translational Medicine Institute, Trinity College Dublin, Dublin, Ireland

**Keywords:** Non-muscle myosin, Actin, Cell migration, Oesophageal adenocarcinoma, Metastasis

## Abstract

The incidence of oesophageal adenocarcinoma (OAC) is increasing at a rapid rate in Western countries. Early oesophageal cancer is often asymptomatic and metastatic disease is common at presentation leading to poor prognosis and survival rates. Cell migration is tightly controlled in the healthy cell but can become dysregulated in diseases such as OAC where increased cell motility and migration can contribute to metastasis. We investigated the role of an actin-based molecular motor, non-muscle myosin heavy chain IIA (NMHCIIA) in the migratory capacity of oesophageal adenocarcinoma cells. Immunofluorescence microscopy and ratiometric imaging demonstrated that NMHCIIA co-localises with F-actin at the leading edge and retracting rear of migrating FLO-1 OAC cells. siRNA-mediated depletion of NMHCIIA from FLO-1 cells altered cell morphology, gave rise to an increased number of stress fibre like structures and reduced FLO-1 cell migration. These findings suggest that NMHCIIA influences FLO-1 cell migration by regulating F-actin dynamics and the actin cytoskeleton, providing insight into the mechanisms of migration employed by OAC cells and identifying NMHCIIA as a potential therapeutic target for this disease.

**Supplementary Information:**

The online version contains supplementary material available at 10.1007/s13577-025-01196-w.

## Introduction

In a worldwide analysis of global statistics for cancer in 2020, oesophageal cancer ranks seventh in terms of incidence and sixth in mortality overall [1]. Despite recent advances in treatment, prognosis for oesophageal cancer is poor, with the overall 5-year survival of patients with this malignancy less than 20% [2]. There are two main histological types of oesophageal carcinoma, namely, oesophageal squamous cell carcinoma (SCC) and oesophageal adenocarcinoma (OAC). On a global level, SCC has the greatest incidence. However, the incidence of OAC is increasing rapidly in the Western world and OAC is now more common than SCC in countries, such as the USA, Australia, and some western European countries [3].

Aberrant regulation of cell migration contributes to the progression of many diseases, including cancer cell invasion and metastasis [4]. The ability of a cancer cell to migrate and invade enables it to change position within tissues and enter lymphatic and blood vessels for dissemination, thus enabling the cancer to colonise distant organs [5]. In many cancers, cell motility and migration is stimulated, and invasiveness enhanced, when a process known as epithelial-to-mesenchymal transition (EMT) occurs [6]. As cells undergo EMT, they lose their epithelial characteristics, such as their polarity and epithelial cell–cell contact structures, and instead acquire a mesenchymal phenotype [6]. They thus acquire a migratory behaviour which allows them to move away from their epithelial cell neighbours and invade surrounding tissue [7]. Cell migration is a dynamic, complex process which requires multiple, interdependent steps. The cytoskeleton must be reorganised to form leading-edge protrusions, mechanical forces must be generated, and the cell tail must be retracted and detached from the extracellular matrix [4]. The mechanisms of cell migration used by migrating cancer cells are similar to those used by healthy cells during normal physiological processes, such as wound healing, immune surveillance, and embryonic morphogenesis [8].

When migration is stimulated, a cell generally polarises and generates cell protrusions in the direction of migration. These protrusions are usually driven by actin polymerization and they vary in nature depending on the cell type; some cells generate broad lamellipodia, whilst others generate spike-like filopodia [9]. Transmembrane receptors linked to the actin cytoskeleton stabilise these protrusions by mediating their adhesion to the extracellular matrix or to adjacent cells. These adhesions act as traction sites for cell movement as the cell progresses forward over them. At the cell rear, adhesions are disassembled, allowing the cell to detach. The collective migration of cell sheets share some of the features of single-cell migration, but in the case of the former, polarisation extends across the sheet [10].

Non-muscle myosin II proteins are actin-binding motor proteins with cross-linking and contractile properties. Their ability to catalyse the conversion of chemical energy into directed movement and force enables them serve as important regulatory components of the cytoskeleton and they are proteins with critical roles in the control of cell adhesion, cell migration, and tissue architecture. Non-muscle myosin II isoforms cyclically interact with adenosine triphosphate (ATP) and F-actin to promote cytoskeletal force generation [11–13]. As a force-producing protein with actin cross-linking properties, non-muscle myosin II is believed to play important roles in cell body translocation and retraction of the posterior of the cell during migration [13–15]. Non-muscle myosin II is a hetero-multimeric protein complex composed of two 230 kDa heavy chains, two 20 kDa regulatory light chains, and two 17 kDa essential light chains. Three mammalian isoforms of non-muscle myosin II have been identified, namely, non-muscle myosin IIA, non-muscle myosin IIB, and non-muscle myosin IIC. These isoforms have both overlapping and distinct functions [11]. Non-muscle myosin II appears to have cell-type specific functions, but it has been found to associate with a wide variety of molecules involved in cell migration, including actin microfilaments, microtubules, S100A4, and cadherin and integrin complexes [12, 13, 16].

This study sought to investigate the role of NMHCII in two OAC cell lines, focussing on its role in cell migration and particularly in the context of cell shape and actin organisation. This is important to elucidate mechanisms of cancer cell migration and invasion.

## Materials and methods

### Cell culture

FLO-1 cells (Culture Collections, Public Health England) and SKGT-4 cells (ATCC) were grown in DMEM or RPMI 1640, respectively, in each case supplemented with 10% FCS and UltraGlutamine and maintained at 37 °C in a 5% CO_2_ humidified atmosphere. FLO-1 was established from a primary distal oesophageal adenocarcinoma in a 68-year-old Caucasian male in 1991. SKGT-4 was established from a primary tumour in 1989 from an 89-year-old Caucasian male who presented with dysphagia secondary to a well-differentiated adenocarcinoma arising in the Barrett’s epithelium of the distal oesophagus. They were both staged as T2N1, meaning that the cancer has grown into the thick muscle wall of the oesophagus with 1–2 local lymph nodes involved (no metastasis). Both cell lines have been whole genome sequenced confirming the presence of many of the known mutations that drive oesophageal cancer [17]. QhTERT and GohTERT cells were kind gifts from Dr Rabinovitch (University of Washington, WA, USA). Both QhTERT and GohTERT cell lines were cultured in BEBM medium with BEGM SingleQuots™ growth supplement kit (Lonza) and 5% FCS. HET1A (ATCC) cells were cultured in BEBM medium with BEGM SingleQuots™ growth supplement kit except GA (gentamycin/amphotericin).

### Cell transfection

FLO-1 and SKGT-4 cells were reverse transfected with 30 µM ON-TARGETplus Non-targeting Control Pool or SMARTpool: ON-TARGETplus MYH9 siRNA (Darmacon) using Lipofectamine RNAiMAX (Invitrogen) at a ratio of 1 µl Lipofectamine: 4 µM siRNA. FLO-1 cells were also forward transfected with 2 µg of a plasmid encoding pCMV-mCherry-MHC-IIA plasmid (Addgene, deposited by Venkaiah Betapudi), or free EGFP (a kind gift from Prof Joel Swanson, University of Michigan) using Fugene HD Transfecting reagent (Promega) at a ratio of 3 µl:1 µg DNA.

### Scratch wound two-dimensional (2D) cell migration assay

A single scratch wound was created in fully confluent monolayers of FLO-1 or SKGT-4 cells using a p200 pipette tip. FLO-1 cells were treated with 50 ng/ml HGF to promote cell migration. Cells were imaged using the Cellavista imaging platform. The size of each wound was quantified at 0, 9, or 24 h using the ‘plaque assay’ tool included in the Cellavista software (Roche, Basel, Switzerland). The area of wound which had been filled by migrating cells was calculated by subtracting the size of the wound at a given post-wounding time point from the original wound area recorded at time zero.

### Methylthiazolyldiphenyl-tetrazolium (MTT) assay

The MTT assay was used to measure cell viability/proliferation in the FLO-1 cell line. Cells were incubated with 0.5 mg/ml methylthiazolyldiphenyl-tetrazolium (MTT) (Sigma) in complete media at 37 °C for 1–3 h. Cells were solubilised in DMSO and the absorbance at 570 nm was measured using an ELx800 Absorbance Plate Reader (BioTek).

### F-actin organisation assay

Cells were fixed with 4% Paraformaldehyde, permeabilized with 0.3% Triton X-100, and blocked with 3% BSA before incubating with phalloidin-TRITC. Cells were imaged using the InCell 1000 High Content Cell Analysis platform. Cells with 4 or more ‘very thick stress fibres’ were counted and expressed as a percentage of total cells in the field of view under analysis. ‘Very thick stress fibres’ were defined as those stress fibres whose width exceeded an arbitrarily set length measured using ImageJ (NIH free download).

### Fluorescent ratiometric imaging of NMHCIIA-mCherry

Cells were maintained at 37 °C on a heated stage in 35 mm MatTek dishes (MatTek Corporation) in Ringer’s buffer (155 mM NaCl, 5 mM KCl, 2 mM CaCl_2_, 1 mM MgCl_2_, 2 mM NaH_2_PO_4_, 10 mM glucose, and 10 mM HEPES at pH 7.2) supplemented with 10% FCS and 2 mM GlutaMAX (Life Technologies). Images were acquired on a Nickon Eclipse TE-300 inverted fluorescence microscope with a 60X 1.4 PlanApo oil immersion objective lens, excitation and emission filters in filter wheels, dichroic mirrors that allowed simultaneous detection of multiple fluorophores, a temperature-controlled stage, shutters for both phase-contrast and epifluorescence illumination, and a cooled digital charge-coupled camera (Quantix; Photometrics). The source of epifluorescent light was a 75 W mercury arc lamp. Images were processed using MetaMorph image processing software (Molecular Devices). Images of chimeric mCherry were collected with 580 nm excitation and 630 nm emission. Images of free EGFP were collected with 485 nm excitation and 525 nm emission. Phase-contrast images were also collected. Ratio images of NMHCIIA-mCherry were obtained by dividing the NMHCIIA-mCherry images by the corresponding free EGFP images. Such ratio images report the localisation of the NMHCIIA-mCherry chimera normalised to cell volume and thus corrected for variation in optical path length due to cell shape. A pseudo-colour scale was applied to the ratio images using imageJ (16_colors table).

### Immunofluorescent staining and confocal microscopy

Cells were fixed with 4% paraformaldehyde, permeabilised with 0.3% Triton X-100, blocked with 3% BSA, and then incubated with phalloidin-TRITC, an anti-NMHCIIA antibody (Cell Signaling Technologies) (1/100 dilution) and AlexaFluor goat anti-rabbit 488 (Molecular probes) (1/200 dilution). Cells were visualised using a Zeiss LSM confocal microscope.

### Sodium dodecyl sulphate–polyacrylamide gel electrophoresis (SDS-PAGE)

Cells were lysed in 10 mM Tris–HCl, pH 7.4, 150 mM NaCl, and 1% Igepal CA-630 (Nonidet P-40) supplemented with freshly added Sigma protease inhibitor cocktail (P8340) (1/100 dilution) and Sigma phosphatase inhibitor cocktail 2 (P5726) and Sigma phosphatase inhibitor cocktail 3 (P0044) both used at a 1/500 dilution. Protein concentrations were determined by BCA assay (Pierce) according to the manufacturer’s protocol. Protein samples were then boiled for 5 min in sample buffer (60 mM Tris–Cl pH 6.8, 2% SDS, 10% glycerol, 5% β-mercaptoethanol, and 0.01% bromophenol blue). Proteins were resolved by SDS-PAGE using the Atto gel electrophoresis system. PVDF membranes were probed with NMHCIIA (Biomedical Technologies), NMHCIIB, NMHCIIC (Cell Signaling Technology), or β-actin primary antibodies and HRP-labelled secondary antibodies (Cell Signaling Technology) in 5% w/v milk powder (Marvel) in 1XTBS-T. Blots were imaged using FUSION gel imaging system (Vilber Lourmat).

### Statistics

Statistical analysis was carried out using Prism v6.0, Prism v7.0 and InStat (all from GraphPad Software, CA, USA). A *P* value ≤ 0.05 was considered significant: *P ≤ 0.05, **P ≤ 0.01, and ***P ≤ 0.001.

## Results

### NMHCIIA is abundantly expressed in SKGT-4 and FLO-1 oesophageal adenocarcinoma cells

**Fig. 1:**
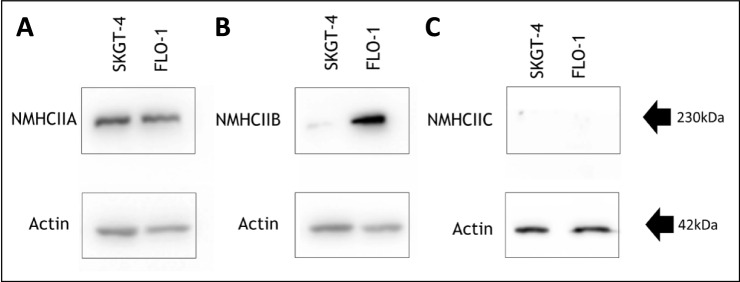
NMHCIIA heavy chain isoform is highly expressed in FLO-1 and SKGT-4 OAC cells. Cell lysates were generated from FLO-1 and SKGT-4 cells and analysed by Western blotting using **A**. NMHCIIA, **B**. NMHCIIB, and **C**. NMHCIIC and β-actin antibodies. Blots are representative of three independent experiments

Three different isoforms of the non-muscle myosin heavy chain exist, namely, non-muscle myosin heavy chain IIA (NMHCIIA), non-muscle myosin heavy chain IIB (NMHCIIB), and non-muscle myosin heavy chain IIC (NMHCIIC). Cancer cells of epithelial origin have varying expression and relative abundance of each non-muscle myosin II heavy chain isoform [18]. Each isoform has different biochemical properties and functions; however, some redundancy between isoforms does exist and it is possible that poorly expressed isoforms may not be as physiologically relevant as those isoforms which are expressed in abundance [19–21]. It was therefore of interest to examine the expression of each NMHCII isoform in OAC cells prior to further examination of the role of NMHCII in cell migration and other functions. Western blotting was used to examine the expression of NMHCIIA, NMHCIIB, and NMHCIIC, and revealed strong protein expression of NMHCIIA in both SKGT-4 and FLO-1 cells (Fig. 1A). Expression of NMHCIIA was also detected (by immunofluorescence) in resting non-cancerous HET1A, Barrett’s metaplastic (QhTERT) and dysplastic GoTERT) cell lines (Supplementary Fig. 1). NMHCIIB expression was also observed in FLO-1 cells, whilst its expression in SKGT-4 cells was barely detectable (Fig. 1B). NMHCIIC expression was not detected in SKGT-4 cells or FLO-1 cells (Fig. 1C). Given that NMHCIIA was highly expressed in both cell lines we were keen to investigate its role in migrating OAC cells.

### NMHCIIA depletion reduces migration of FLO-1 cells but does not affect migration of SKGT-4 cells in a wound-healing assay

**Fig. 2:**
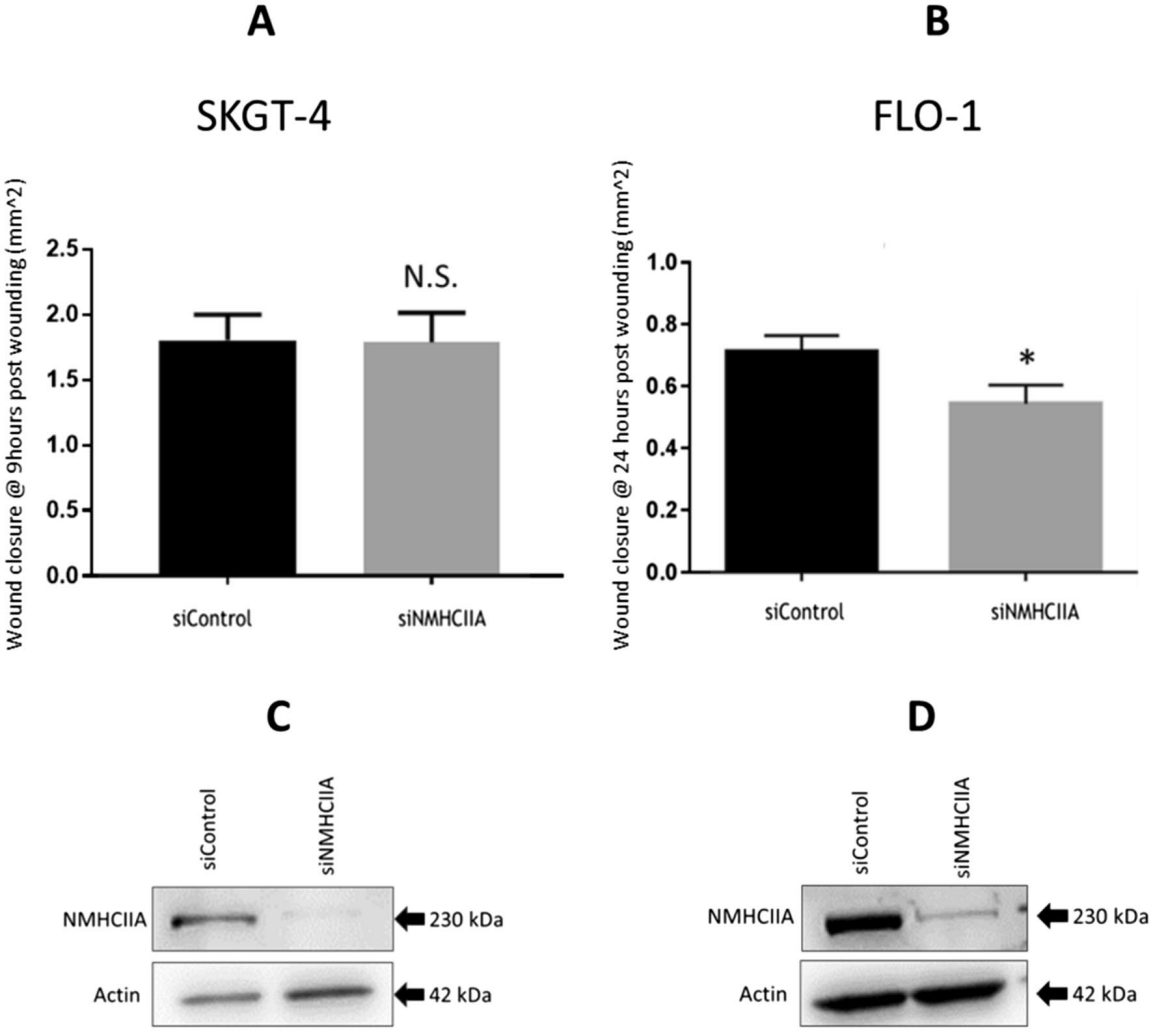
NMHCIIA depletion reduces FLO-1 cell migration but does not reduce SKGT-4 cell migration. SKGT-4 or FLO-1 cells were transfected with siRNA specific for NMHCIIA or a control siRNA. Cells were allowed to form a confluent monolayer in which a scratch wound was made. Migration into the wound was assessed by measuring the area of wound closure after (**A**) 9 h for SKGT-4 or (**B**) 24 h for FLO-1 cells. N.S. not significant; *P ≤ 0.05, unpaired t test. Data presented are mean ± SEM of three independent experiments. (**C**, **D**) Lysates were also generated and analysed by Western Blotting using NMHCIIA and β-actin antibodies. Blots are representative of three independent experiments

Given the conflicting roles of NMHCIIA in cancer cell migration, we sought to determine the role of NMHCIIA in OAC cell migration. To investigate this, FLO-1 and SKGT-4 were transiently transfected with siRNA to deplete cells of NMHCIIA before assessing their ability to migrate using a scratch wound assay. Approximately 80% depletion of protein expression was observed in each cell line, which was confirmed by measuring protein levels of NMHCIIA (Fig. 2C, D). SKGT-4 cells migrate collectively in cell sheets and do not display visible individual lamellipodia. Conversely FLO-1 cells form pseudopod structures when scratch wounded and treated with hepatocyte growth factor (HGF) [22]. Interestingly, we observed no reduction in migration in NMHCIIA-depleted SKGT-4 cells (Fig. 2A). Using this experimental design, we observed a significant reduction in migration when FLO-1 cells were depleted of NMHCIIA (Fig. 2B). Together, these findings indicate that NMHCIIA plays an important role for single-cell migration of OAC cells.

### NMHCIIA co-localises with F-actin at the lamellipodial leading edge of migrating FLO-1 cells but does not co-localise with F-actin at the leading edge of migrating SKGT-4 cells

**Fig. 3:**
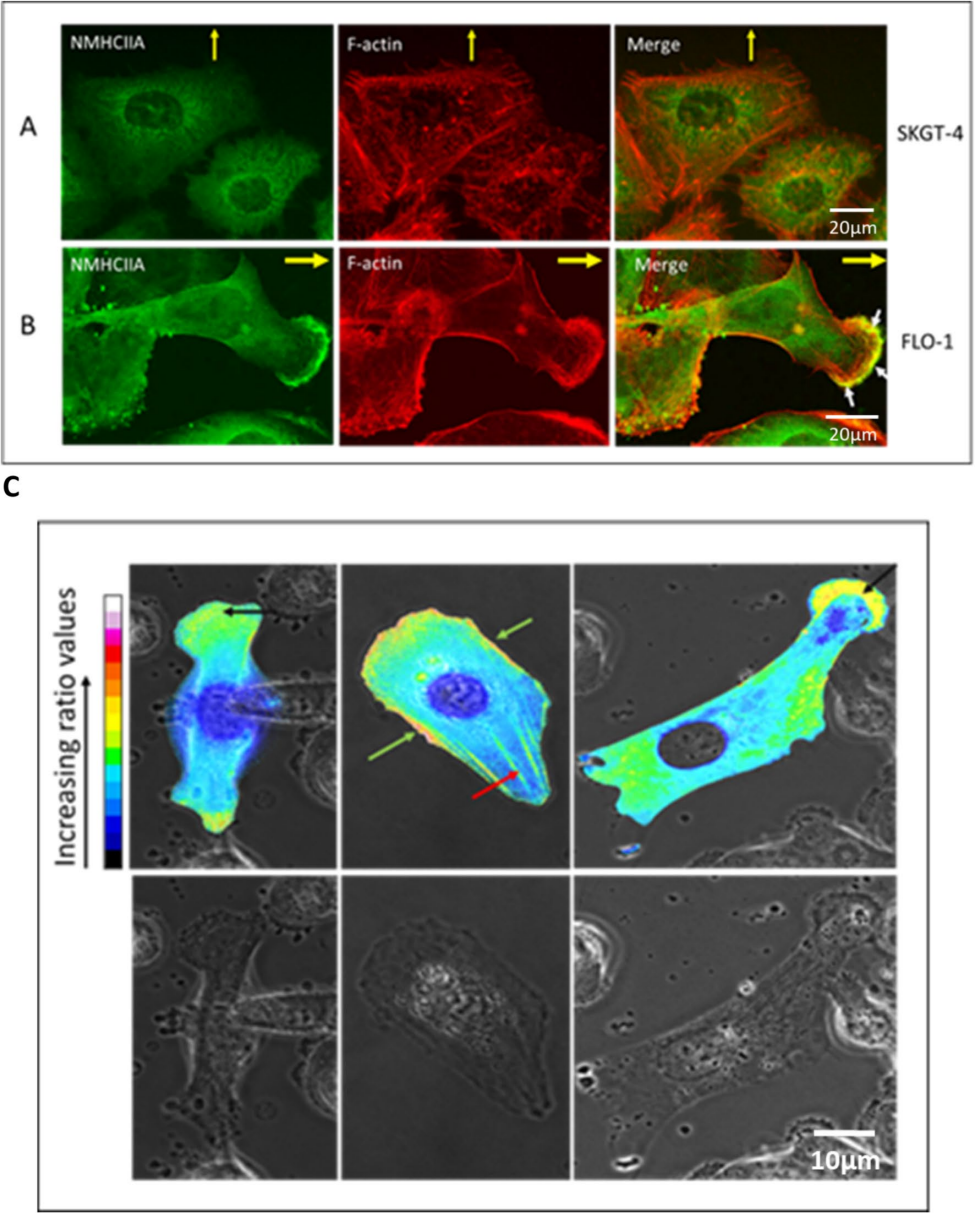
NMHCIIA co-localises with F-actin at the lamellipodial leading edge of migrating FLO-1, but not SKGT-4, cells. SKGT-4 cells (**A**) or FLO-1 cells (B) were grown to confluence and a scratch wound was then made in the cell monolayer. At 24 h post-wounding, cells were fixed, permeabilised, and stained with phalloidin and immunofluorescent dye conjugated to an antibody specific for NMHCIIA. Images were collected by confocal microscopy using a 63X objective. Yellow arrows indicate direction of migration into scratch wounds. White arrows indicate co-localisation. **C** mCherry tagged NMHCIIA localises to the leading edge and to ruffling lamellipodia in live, migrating FLO-1 cells. Images representing ratios of mCherry-NMHCIIA to EGFP fluorescence were produced. mCherry-NMHCIIA fluorescent signal was thus normalised to the distribution of the cytosol, using EGFP. These ratiometric images were overlaid onto phase-contrast images (top panels). Subcellular regions to which NMHCIIA localised could thus be identified by identifying regions where the ratio of mCherry to EGFP was high. High ratio values observed at the leading edge of migrating cells and in membrane ruffles are indicated by black arrows. High ratio values observed at the cell periphery are indicated by green arrows. Stress fibres are highlighted with red arrow. Bottom panels display phase-contrast images alone and enable the morphology of the migrating cells and positioning of lamellipodial ruffles to be clearly seen

Given that suppression of NMHCIIA inhibited migration of FLO-1 cells, we were keen to further investigate its role in FLO-1 cell migration. We therefore sought to analyse its localisation in relation to leading-edge F-actin during migration of both FLO-1 and SKGT-4 cells. Migrating cells were therefore co-stained with immunofluorescently tagged phalloidin (which selectively binds F-actin) and immunofluorescent dye conjugated to an antibody specific for NMHCIIA. This revealed that NMHCIIA co-localises with F-actin at the lamellipodial leading edge of migrating FLO-1 cells (Fig. 3B). SKGT-4 cells do not display visible lamellipodia, but a ‘leading edge front’ can be identified which faces in the direction of migration; strong F-actin staining was observed at this leading front and was also observed around most of the cell periphery. We observed no NMHCIIA co-localisation with F-actin at the leading-edge front of SKGT-4 cells (Fig. 3A); expression of NMHCIIA was concentrated around the nucleus. Thus, we have observed that NMHCIIA co-localises with F-actin at the leading edge of FLO-1 cells and this protein is required to maintain the migration rate of FLO-1 cells. In contrast, NMHCIIA does not co-localise with F-actin in SKGT-4 cells, whose rate of migration is not reduced when they are depleted of this protein.

Live-cell ratiometric imaging was then used to further confirm the cellular localisation of NMHCIIA during FLO-1 migration. NMHCIIA-mCherry was co-expressed with free EGFP fluorescent protein, a marker of cytoplasmic volume, in FLO-1 cells and corrected for variation in optical path length and fluorescent intensity due to variation in cell shape and thickness. Three types of image were collected and used to produce ratiometric images; phase contrast, mCherry, and EGFP. Images representing ratios of mCherry to EGFP fluorescence were produced and overlaid onto the phase-contrast images. The mCherry signal was thus normalised to the distribution of the cytosol, using EGFP. Subcellular regions to which NMHCIIA localised could thus be identified by identifying regions where the ratio of mCherry to EGFP was high. Scratch wounding and treatment with HGF was employed to stimulate FLO-1 cell migration. Cells which exhibited membrane ruffling and a polarised morphology consistent with cell migration were identified for analysis. In such cells, NMHCIIA was most concentrated at the leading edge where it was particularly concentrated in membrane ruffles (Fig. 3C, black arrows). High NMHCIIA-mCherry concentration was also observed at the cell periphery (green arrows) and in stress fibres (red arrows, Fig. 3C).

### NMHCIIA depletion alters FLO-1 cell morphology and F-actin organisation

**Fig. 4:**
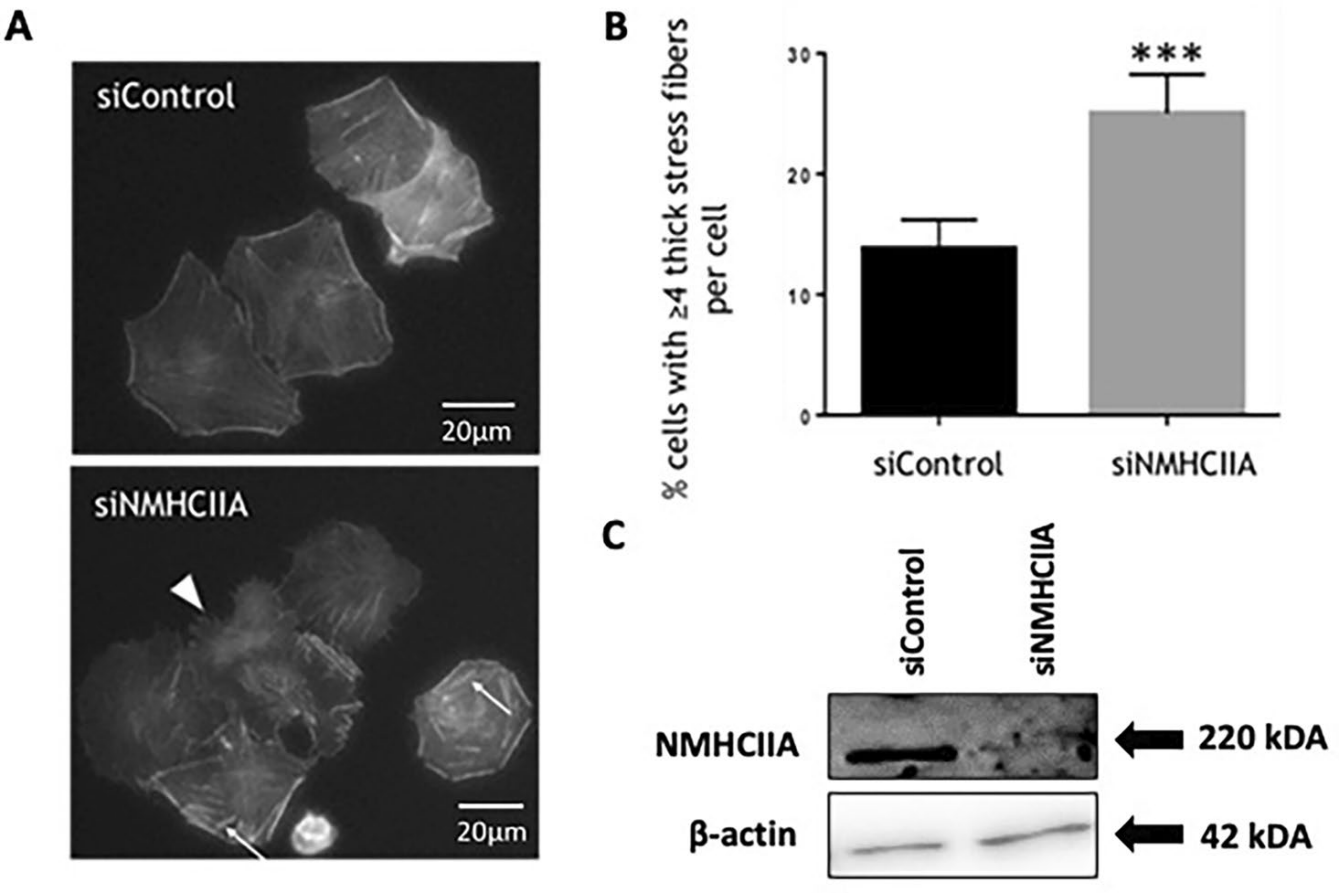
NMHCIIA depletion alters FLO-1 cell morphology and F-actin organisation. FLO-1 cells were transfected with siRNA specific for NMHCIIA or the control siRNA. **A** Cells were fixed and stained with Phalloidin. Stress fibres are indicated with white arrows and altered morphology with ragged cell edges is highlighted by the white arrow head. **B** The percentage of cells with  ≥ 4 thick stress fibres was determined. Data presented are mean ± SEM of three independent experiments. ***P ≤ 0.001, unpaired Student’s t test. **C** Lysates were generated and analysed by Western blotting using NMHCIIA and β-actin antibodies. Blot is representative of the three independent experiments

Since NMHCIIA co-localises with F-actin in FLO-1 cells, we hypothesised that NMHCIIA may play a role in regulating or organising F-actin structures in this cell type. To test this, we depleted FLO-1 cells of NMHCIIA using siRNA before using phalloidin to stain F-actin. Knockdown of NMHCIIA has been shown not to affect expression or function of NMHCIIB in multiple model systems [23–26]. Interestingly, NMHCIIA-depleted FLO-1 cells displayed altered cell morphology and a ‘ragged’ cell periphery (highlighted by a white arrowhead, Fig. 4A). NMHCIIA-depleted cells also had an increased number of very thick, F-actin-rich stress fibres (highlighted by the white arrows, Fig. 4A). Cells displaying four or more of these thick stress fibres were counted and expressed as a percentage of total cells in the population. This demonstrated that the NMHCIIA-depleted population of cells had a statistically significant increase in the percentage of cells with ≥ four thick stress fibres (Fig. 4B). These results indicate that NMHCIIA is required for the maintenance of normal F-actin dynamics in FLO-1 cells. NMHCIIA may be required to regulate F-actin bundling, to control the size and integrity of stress fibres, or to promote the efficient turnover of stress fibres. Thus, the reduced rate of migration observed in NMHCIIA-depleted FLO-1 cells is likely due to altered F-actin dynamics and organisation.

### NMHCIIA depletion does not affect FLO-1 cell proliferation as assessed by MTT assay

**Fig. 5:**
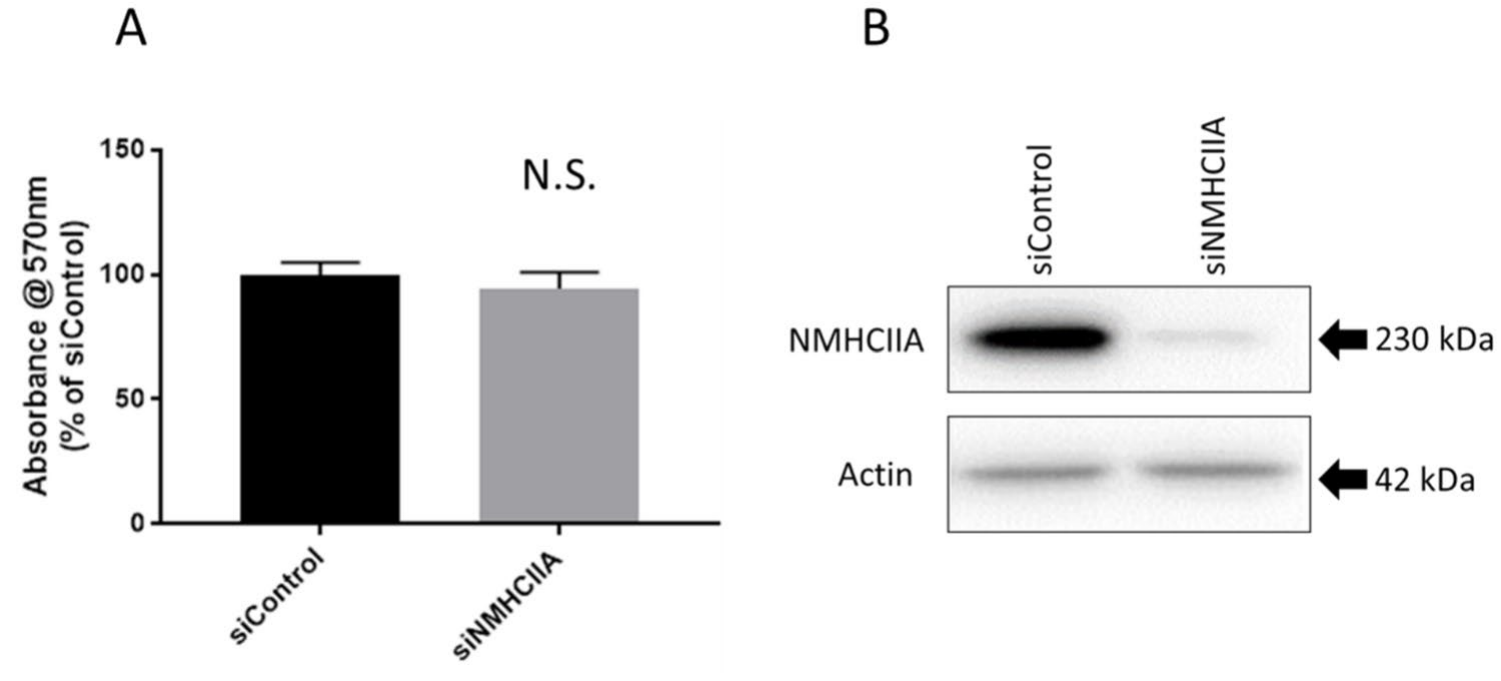
NMHCIIA depletion does not affect FLO-1 cell proliferation. FLO-1 cells were transfected with siRNA specific for NMHCIIA or the siRNA control. **A** Cell proliferation was assessed by MTT assay. Data presented are mean ± SEM of four independent experiments. N.S. Not Significant, unpaired Student’s t test. **B** Lysates were generated and analysed by Western blotting using NMHCIIA and β-actin antibodies. Blot is representative of the four independent experiments

We also examined the impact of NMHCIIA depletion on FLO-1 cell proliferation to determine if the inhibitory effects of NMHCIIA on FLO-1 cell migration were due to reduced cell viability or proliferation. Therefore, an MTT assay was employed to assess the proliferation of FLO-1 cells depleted of NMHCIIA. We found no difference in proliferation between NMHCIIA-depleted FLO-1 cells when compared with FLO-1 cells transfected with the siRNA control (Fig. 5A). The successful depletion of NMHCIIA was confirmed by Western blotting (Fig. 5B). This indicates that the NMHCIIA depletion induced decrease in FLO-1 cell migration that is reported is independent of proliferation-associated processes and more likely a result of altered F-actin organisation.

## Discussion

The three non-muscle myosin isoforms exhibit a high degree of sequence similarity at the amino acid level. However, each isoform does have different enzymatic and biochemical properties, and thus, they can have both interchangeable and unique functions [11, 13]. The relative expression of each NMHCII isoform varies with different cell and tissue types, with some cells/tissues expressing all three isoforms in varying proportions, whilst other cells/tissues express only one or two of the three isoforms [18]. NMHCIIA was highly expressed in both SKGT-4 and FLO-1 OAC cells. Intriguingly, NMHCIIB expression was stronger in FLO-1 cells than SKGT-4 cells and NMHCIIC expression was not detected in either cell line. This finding illustrates that the profile of NMHCII isoform expression varies not only between cells isolated from different organs but also between cancer cells isolated from the same cancer type. It is possible that this variation in expression might, at least in part, account for the sometimes-conflicting reports with regard to the role of NMHCII isoforms in cancer. NMHCIIA and IIB have been shown to play distinct roles in the intrinsic and directed migration of human embryonic lung fibroblasts and also in murine and human tumourigenic cells [27–29]. We demonstrate here that NMHCIIA is abundantly expressed by both SKGT-4 and FLO-1 cells, highlighting the importance of this isoform in OAC cells.

The differential role of NMHCIIA in cell migration in FLO-1 and SKGT-4 cells, reflected by distinct subcellular distribution, may explain the different modes of migration in the two cell lines. In vivo, cancer cells can use a variety of migration modes to achieve successful spreading and metastasis. These include collective cell migration, which has been observed in many tumours of epithelial origin that still have cadherin-based cell–cell junctions, and single-cell migration which is characteristic of leukaemia cells, lymphoma cells, and cells that have originated from solid tumours that have undergone EMT and lost cadherin expression [18, 30]. Interestingly, in further characterisation of the cell lines, FLO-1 has undergone epithelial–mesenchymal transition and metastasizes following subcutaneous injection in mice [31], whereas the SKGT-4 cell line has been shown to exhibit minimal invasive potential [32].

In our hands, SKGT-4 cells migrate collectively, in a tight cell sheet in which contacts between cells are retained. NMHCIIA displays a peri-nuclear localisation and minimal colocalization with actin. In scratch wound assays, FLO-1 cells also exhibit some collective cell migration, but we also observed single-cell migration, whereby they separated from their neighbours during migration into a scratch wound. We could identify individual lamellipodia and a clear front-rear polarity in FLO-1 cells with NMHCIIA and actin colocalising at the leading lamellipodium. We observed no visually detectable lamellipodia in SKGT-4 cells. It has been proposed that myosin II molecules might also contribute to membrane trafficking events related to leading-edge functions. Indeed, myosin II has been associated with vesicle trafficking in a number of settings which could be relevant to leading-edge functions such as the delivery of adhesion molecules or signalling proteins during migration [14, 26, 33].

During single-cell migration, the principal engine for movement appears to be at the cell front, where dynamic membrane protrusion occurs, and the cell adheres to the extracellular matrix. As the lamellipodium extends forward, new integrin-based adhesions form which eventually mature into focal adhesions that serve as anchors for longitudinal acto-myosin cables. These mature focal adhesions are retained until they reach the retracting edge of the cell, where they undergo disassembly [34]. Myosin activity is undoubtedly important for many types of cell migration. Single cell migration is highly reliant on cell contractility for which myosin activity is likely to be particularly important. As our observed differences between SKGT-4 and FLO-1 cells illustrate, the importance of particular forms and isoforms of myosin for migration can vary between cells and is likely to depend on the particular mode of migration employed by a cell. Tumour cells can exhibit plasticity in their ability to switch between forms of migration (which makes treatment to inhibit migration/metastasis more difficult) and differential regulation of myosin isoforms may be important for selection of a particular migration mode [18].

In addition to reducing migration, we observed that NMHCIIA depletion also alters the morphology of FLO-1 cells and gives the cell a ‘ragged’ cell periphery. Furthermore, we have found that NMHCIIA depletion leads to an increase in thick, F-actin-rich stress fibres, in FLO-1 cells. This suggests that NMHCIIA is required to maintain normal F-actin dynamics such as F-actin bundling or regulation of stress fibres. Thus, the reduced rate of migration observed in FLO-1 cells depleted of NMHCIIA may be due to disrupted F-actin dynamics and organisation. A study by Hindman and colleagues examined the effects of NMHCIIA depletion on cell morphology and actin organisation in MDA-MB-231 breast carcinoma cells [35]. As we had observed in FLO-1 cells, NMHCIIA depletion disrupted the normal organisation of actin stress fibres in MDA-MB-21 cells. However, whilst we observed an increase in thick stress fibres throughout the cell, Hindman et al*.* found that NMHCIIA depletion from MDA-MB-321 cells resulted in an increase in prominent stress fibres at the periphery of the cell and a decrease in centrally located fibres*.* Interestingly however, the Hindman study also found that NMHCIIB depletion from MDA-MB-231 cells resulted in the formation of thicker, shorter stress fibres in MDA-MB-231 cells. The mechanism of how NMHCIIA affects cell migration in FLO-1 cells is not fully understood. However, using a glioma cell model, a study by Chang and Kumar analysed how Non-Muscle Myosin II isoforms, and more specifically how functional domains of these isoforms, contribute to stress fibre mechanics [23]. They found NMHCIIA enriched in central stress fibres and co-localised with Rho Kinase (ROCK), whereas NMHCIIB associates with myosin light-chain kinase (MLCK) enriched at peripheral fibres. They propose a model in which NMHCIIA cross-linking and motor functions jointly contribute to stress fibre retraction dynamics and cellular traction forces. The specific regulation of NMHCIIA by ROCK has been confirmed and characterised in other cell types including epithelial cells [36, 37].

Kuragano et al. investigated roles for NMHCIIA and IIB in formation of stress fibre subtypes transverse arcs and ventral stress fibres. In knockdown experiments, NMHCIIB could rescue defects in transverse arcs but not lamellar flattening caused by NMHCIIA. On the other hand, NMHCIIA expression could not rescue loss of ventral stress fibres caused by knockdown of NMHCIIB, demonstrating non-redundant roles for the two isoforms in stress fibre formation [24]. A study using lung carcinoma cell lines found that NMHCIIA depletion from A549 cells caused them to become almost devoid of stress fibres, although they did occasionally display some bundled F-actin [38]. In these cells, NMHCIIA depletion also gave rise to a more motile phenotype with a quicker rate of migration in scratch wound assays; thus, these findings are almost in direct contrast to our findings in FLO-1 cells and may be due to differences between the two cell types. Meanwhile, results from a study in colonic adenocarcinoma cells (SK-CO15) were similar to our findings in FLO-1 cells, in that depletion of NMHCIIA reduced cell migration into a scratch wound and gave rise to cells with a ragged cell morphology. However, in contrast to our findings, NMHCIIA-depleted SK-CO15 cells displayed diffuse unstructured F-actin labelling and no prominent stress fibres were visible [19].

A study investigated NMHCIIA expression in oesophageal squamous carcinoma patient tissue (and compared to normal adjacent control tissue) identified this protein to be increased in cancer and to correlate with increasing metastatic lymph nodes, poorer cancer differentiation, advanced tumour stage, and poorer overall survival [39]. Furthermore, using a siRNA approach in a squamous cell carcinoma cell line (KYSE-510), these researchers demonstrated expression of NMHCIIA to mediate increased 2- and 3-D cancer cell migration. Gene expression profile analysis of oesophageal squamous cell carcinoma tissue showed that the gene coding for NMHCIIB (MYH10) was increased relative to control normal tissue [40]. The same study identified genes associated with lymph-node metastasis and MYH10 was not listed.

In conclusion, we have shown that both oesophageal adenocarcinoma cell lines FLO-1 and SKGT-4 express the motor protein NMHCIIA. However, NMHCIIA co-localises with actin at lamellipodia in the FLO-1 cells migrating as single cells with no colocalization, or expression at lamellipodia, in the SKGT-4 cell line migrating as a sheet. Depletion of NMHCIIA in FLO-1 cells alters cell morphology and leads to an increased number of thick, F-actin-rich stress fibres. This suggests that NMHCIIA may be required to maintain normal F-actin dynamics in these cells and that the reduced rate of migration observed in NMHCIIA-depleted FLO-1 cells may be due to disrupted F-actin organisation. NMHCIIA might therefore play a role in the migration of OAC cells which have undergone EMT and therefore may promote cancer cell invasion of surrounding tissues and distal organs.

## Supplementary Information

Below is the link to the electronic supplementary material.

Supplementary file1 (DOCX 581 KB)
