# Supplementary material for: Non-muscle myosin heavy chain IIA regulates cell morphology, stress fibre structure, and cell migration in FLO-1 oesophageal adenocarcinoma cells
Source: Hum Cell. 2025 Mar 31;38(3):80. doi: 10.1007/s13577-025-01196-w (PMC11958448; doi:10.1007/s13577-025-01196-w)
Supplement: Supplementary file 1 — Supplementary file1 (DOCX 581 KB) [file 13577_2025_1196_MOESM1_ESM.docx]

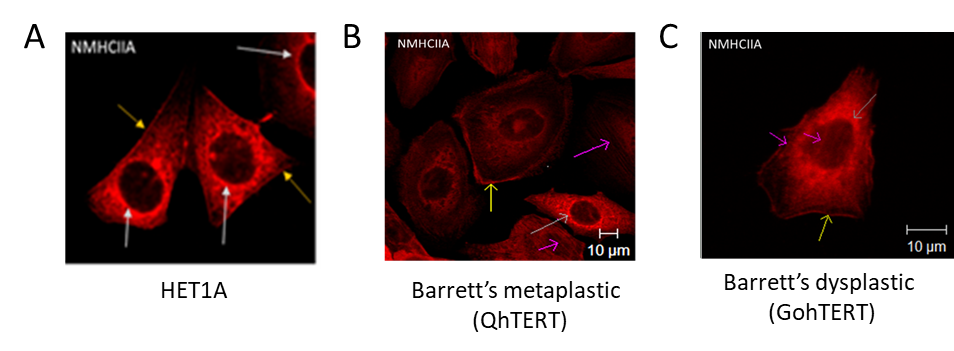


**Supplementary Figure 1: Subcellular localisation of RACK1 and NMHCIIA in resting non-cancerous, metatastic and dysplastic oesophageal cells.** (A) HET1A, Barrett’s (B) metaplastic and (C) dyplastic cells were fixed, permeabilised, and stained with an antibody specific NMHCIIA conjugated with an immunofluorescent dye. NMHCIIA exhibited a diffuse cytoplasmic distribution which was more concentrated in the perinuclear area of many cells (grey arrow). Strong NMHCIIA staining was also observed at the edge of many cells (yellow arrows) where it may be localised to peripheral stress fibers. NMHCIIA staining was also observed in stress fiber-like structures (pink arrows).


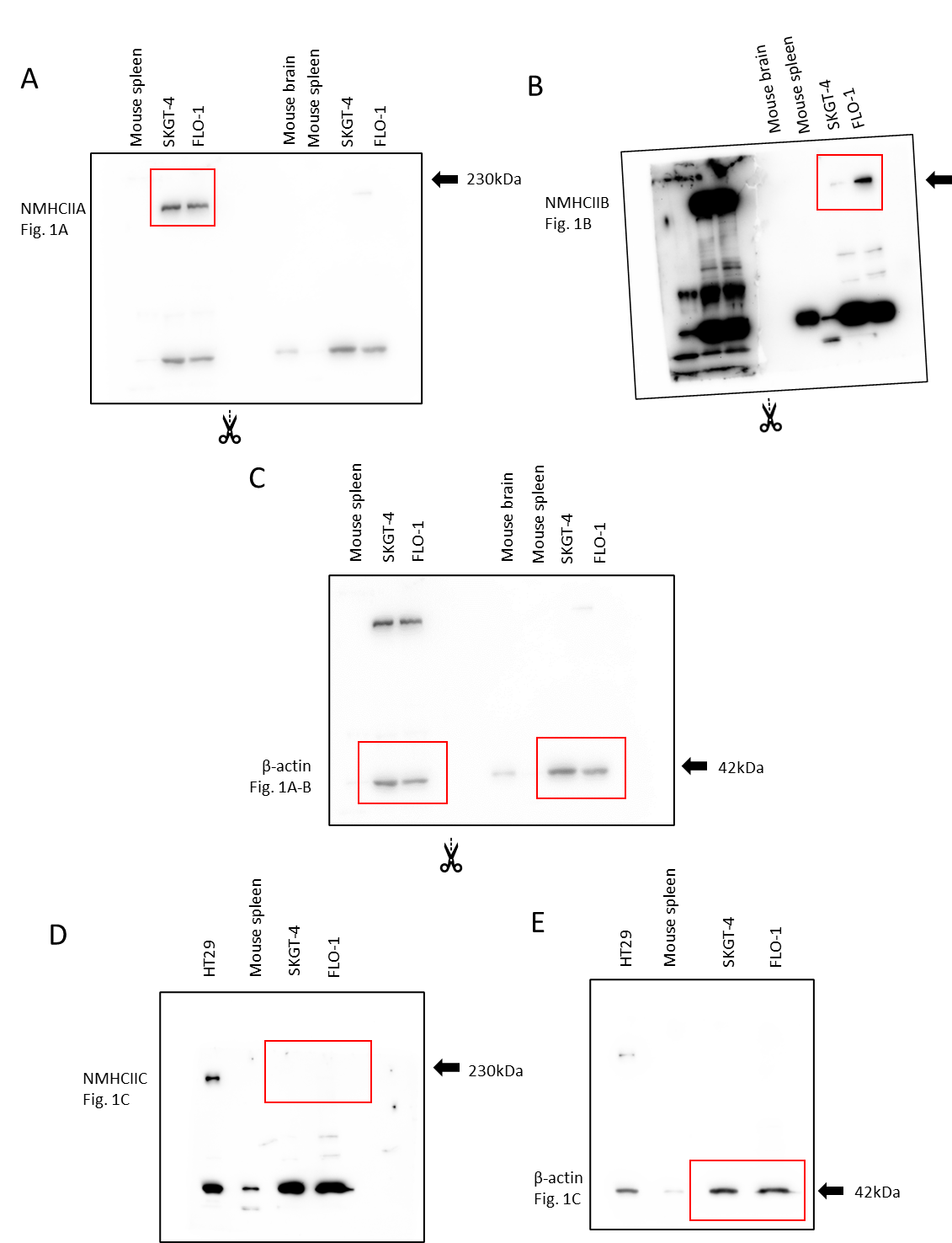


**Supplementary Fig. 2. Full western blot images for Fig. 1.** Protein lysates generated from SKGT-4 and FLO-1 cells were separated by SDS-PAGE gel electrophoresis alongside mouse brain and mouse spleen positive controls (not included in manuscript). Proteins were transferred to a PVDF membrane which was cut as indicated and probed for either (A) NMHCIIA or (B) NMHCIIB and (C) β-actin. Protein lysates generated from SKGT-4 and FLO-1 or HT29 cells and mouse spleen (positive control) were analysed by western blotting using (D) NMHCIIC and (E) β-actin antibodies.


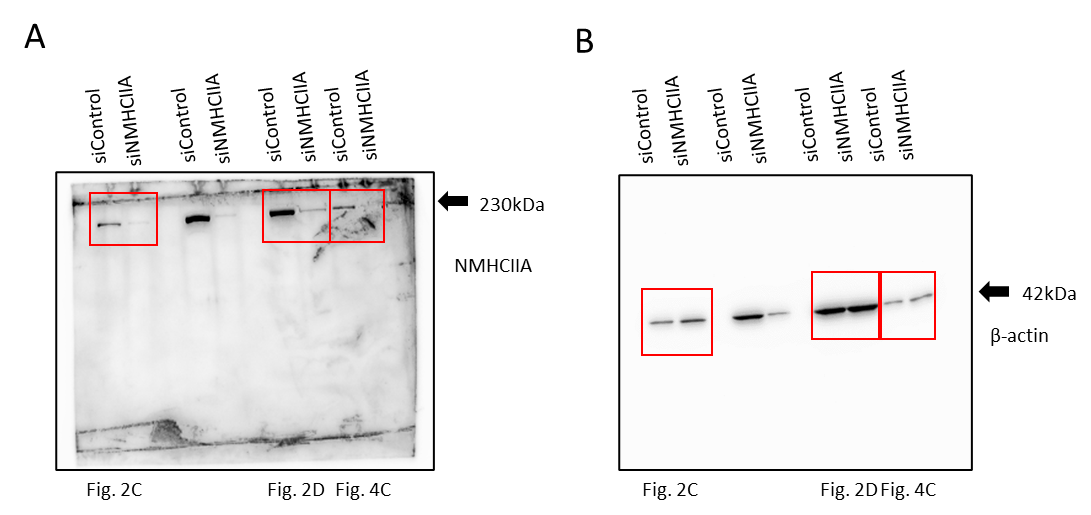


**Supplementary Fig. 3. Full western blot images for Fig. 2 and 4.** FLO-1 cells were transfected with siRNA specific for NMHCIIA or the siRNA control. Lysates were generated and analysed by western blotting using (A) NMHCIIA and then (B) β-actin antibodies.
